# Wrapping Pathways of Anisotropic Dumbbell Particles by Giant Unilamellar Vesicles

**DOI:** 10.1021/acs.nanolett.3c00375

**Published:** 2023-05-04

**Authors:** Ali Azadbakht, Billie Meadowcroft, Thijs Varkevisser, Anđela Šarić, Daniela J. Kraft

**Affiliations:** † Soft Matter Physics, Huygens-Kamerlingh Onnes Laboratory, Leiden University, PO Box 9504, 2300 RA Leiden, The Netherlands; ‡ Department of Physics and Astronomy, Institute for the Physics of Living Systems, University College London, London WC1E 6BT, United Kingdom; § Institute of Science and Technology Austria, 3400 Klosterneuburg, Austria; ∥ Van der Waals-Zeeman Institute, Institute of Physics, University of Amsterdam, Science Park 904, 1098 XH Amsterdam, Netherlands

**Keywords:** colloids, lipid membranes, ligand−receptor interactions, endocytosis, engulfment

## Abstract

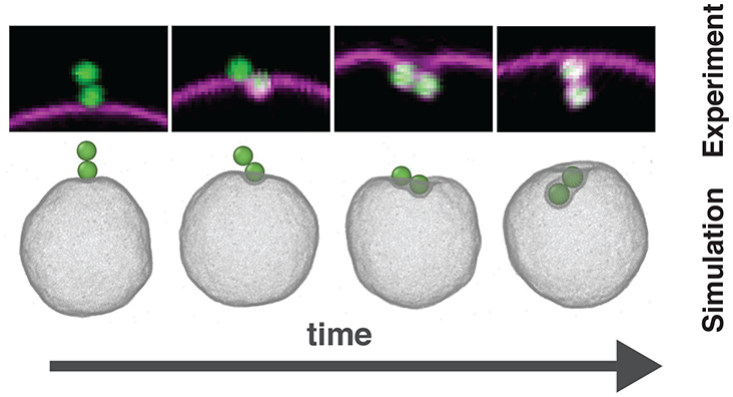

Endocytosis is a key cellular process involved in the uptake of nutrients, pathogens, or the therapy of diseases. Most studies have focused on spherical objects, whereas biologically relevant shapes can be highly anisotropic. In this letter, we use an experimental model system based on Giant Unilamellar Vesicles (GUVs) and dumbbell-shaped colloidal particles to mimic and investigate the first stage of the passive endocytic process: engulfment of an anisotropic object by the membrane. Our model has specific ligand–receptor interactions realized by mobile receptors on the vesicles and immobile ligands on the particles. Through a series of experiments, theory, and molecular dynamics simulations, we quantify the wrapping process of anisotropic dumbbells by GUVs and identify distinct stages of the wrapping pathway. We find that the strong curvature variation in the neck of the dumbbell as well as membrane tension are crucial in determining both the speed of wrapping and the final states.

The engulfment of objects through the cell membrane is critical for endocytic processes such as phagocytosis^1−3^ and receptor-mediated endocytosis. The latter is often exploited by viruses for cell entry and proliferation^4^ and key to nanomedical applications such as drug delivery and imaging.^5^ To single out receptor-mediated effects from active mechanisms involved in the engulfment,^6^ simplified passive model systems can be employed, which recently led to a conclusive understanding of the wrapping of spherical objects.^7,8^ However, biological objects such as bacteria and viruses^4,9,10^ as well as nanoparticles relevant for applications in nanomedicine but also nanotoxicology^11^ often possess nonspherical shapes. Moreover, *in vitro* experiments with nanoparticles and simulations have shown that the size and shape influence their likelihood to be taken up by endocytosis.^6,12−17^

The wrapping pathways of spheres at sufficiently low membrane tensions have been shown to be a continuous transition from attached to fully wrapped, occurring either spontaneously or after activation.^7,8,18^ In contrast, anisotropic particles such as ellipsoids and rods are expected to reorient during the wrapping process or become trapped in metastable states due to their varying curvature.^19−27^ The aspect ratio of these particles as well as the degree of rounding of their tip were the key parameters affecting the wrapping orientation with respect to the membrane and their metastable and stable states.^24,27^ Despite the extensive work in theory and simulations and exciting observations on shape-dependence in phagocytosis,^28^ no experimental work has investigated the passive wrapping process of anisotropic particles by lipid membranes and tested these predictions yet.

In this letter, we employ an experimental model system based on Giant Unilamellar Vesicles (GUVs) and colloidal dumbbell particles to investigate the wrapping of micrometer-sized anisotropic objects by lipid membranes. Our model system is designed to have mobile ligands on the vesicles and immobile receptors on the particles mimicking receptor-mediated endocytotic systems.^18,29,30^ We quantify the wrapping pathways of anisotropic dumbbells by lipid membranes and test if their initial orientation affects the final states. Molecular dynamics simulations of the same system corroborate our experimental data, allowing us to inspect the dynamics of the process that was inaccessible to experiment. We find that the strong curvature variation in the neck of the dumbbell as well as membrane tension and not their initial orientation are crucial in both determining the speed of wrapping and the final states.

We investigate the wrapping process of anisotropic objects by a lipid membrane using a model system consisting of GUVs and colloidal particles (see Figure 1a). We chose the simplest object that features anisotropy: a dumbbell shaped colloidal particle that consists of two equal sized spheres. The colloid dumbbells were obtained from aggregating polystyrene spheres with diameter *d*_*s*_ = 0.98 ± 0.03 μm^31^ by briefly lowering the pH to 5.3 and then quenching the process by increasing the pH to 8.6.^32^ This process yielded 5–10% dimers with a long axis of 1.96 ± 0.06 μm and a short axis of 0.98 ± 0.03 μm. The spherical particles that make up the dumbbells have previously been used in similar experiments,^18^ where it was found that increasing the adhesion energy increases the percentage of wrapped particles and that increasing the membrane tension precluded wrapping. In the current system we functionalized the particles with the highest concentration of ligands explored in ref (18) to enhance the probability of wrapping. GUVs were prepared by electroswelling from 97.5% w/w 1,2-dioleoyl-*sn*-glycero-3-phosphocholine (DOPC).

**Figure 1:**
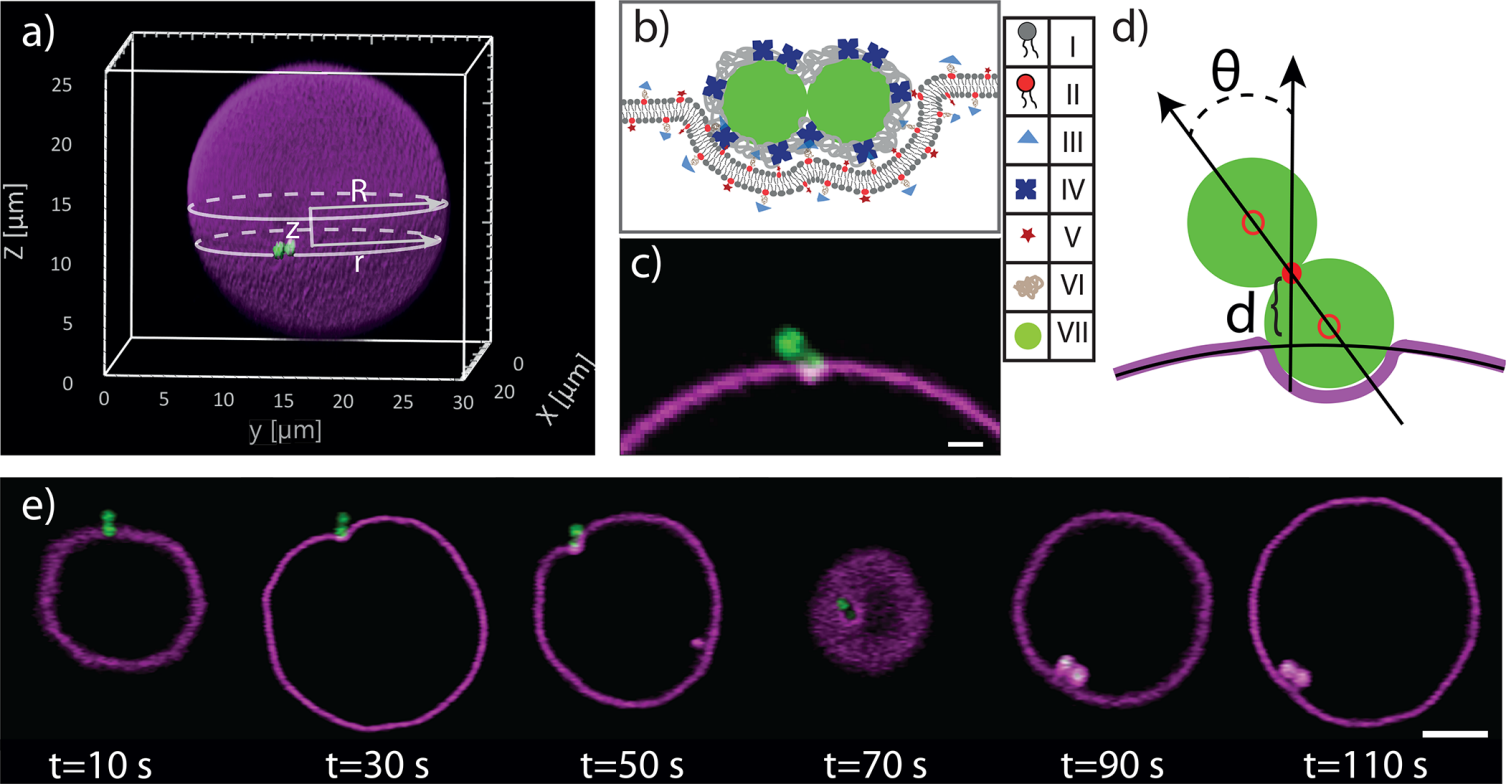
Experimental setup to quantitatively measure the wrapping process of a dumbbell colloid by a GUV. (a) 3D confocal reconstruction of a GUV in magenta and a dumbbell particle in green with an indication of the relative height *z* from the equator of the GUV, radius of GUV *R*, and cross-sectional radius of the vesicle at the location of the dumbbell, *r*. (b) Detailed schematic of ligand–receptor based binding scheme between the dumbbell and GUV. I, DOPC lipid; II, DOPE lipid; III, biotin; IV, NeutrAvidin; V, Rhodamine; VI, polyethylene glycol (PEG); VII, polystyrene particle (Not to scale). (c) Representative confocal images reconstructed from two channels: (1) dumbbell excited by 488 nm laser light and emission collected between 500 and 550 nm (depicted in green) and (2) GUV excited by 561 nm laser light and emission collected in 580−630 nm (depicted in magenta) (scale bar 1 μm). (d) Schematic representation of the parameters *d* and θ used for the quantitative description of the wrapping process. (e) Time series of snapshots of confocal images of a dumbbell being wrapped by a vesicle (scale bar 4 μm).

To realize strong ligand–receptor mediated binding we doped the GUVs with 2% w/w 1,2-dioleoyl-*sn*-glycero-3-phosphoethanolamine-*N*-[biotin-2000] (DOPE-PEG2000-Biotin) and the dumbbells with 2.2 × 10^3^/μm^2^ NeutrAvidin following ref (31); see Figure 1b,c and particle functionalization and quantification of binding affinity in the Supporting Information. We suppress electrostatic interactions by working in 50 mM phosphate-buffered saline (PBS) and achieve colloidal stability by coating the dumbbells with polyethylene glycol (PEG5000). Imaging of the position and orientation of the dumbbells and membranes in three dimensions was made possible by dying the colloids with BODIPY, represented by a green color throughout the manuscript, as well as including 0.5% w/w 1,2-dioleoyl-*sn*-glycero-3-phosphoethanolamine-*N*-(lissamine rhodamine B sulfonyl) (DOPE-Rhodamine) into the GUVs, represented by a magenta color; see Figure 1c. Confocal stacks and image sequences were acquired with an inverted Nikon TI-e microscope, equipped with a 60× (NA 1.2) objective and A1-R scan head. 2D image sequences were taken at 59 fps, which enables tracking of the dumbbells in real time. Experimental details are described in the Supporting Information.

To initiate the wrapping process, we used optical tweezers to bring dumbbell particles in contact with the GUV. They subsequently diffused on the GUV surface before suddenly and quickly becoming wrapped, a process that took between a few seconds and a few minutes depending on membrane tension; see Figure 1e and Movie S1. To capture the wrapping process with high speed, we adjusted the focal height during acquisition of the image sequence. After wrapping, the dumbbell continued to diffuse on the inside of the vesicle.

We quantify the wrapping process of a dumbbell by measuring the angle θ between the major axis of the dumbbell and surface normal of the GUV and distance *d* of the dumbbell with respect to the undistorted surface of the GUV; see Figure 1d. We inferred the 3D position of the dumbbell from the position of its lobes with respect to the GUV. To improve the accuracy of tracking, particles were tracked only when their center of mass was between −0.8*R* < *z* < 0.8*R* and when both lobes were in focus. Details are described in the Supporting Information.

We show confocal microscopy snapshots of a typical wrapping pathway in Figure 1e, and quantitative data of θ and *d* for exemplary pathways in Figure 2a,b. Surprisingly, we find that the dumbbells end up in one of two states, independent of their initial orientations: either (1) both lobes are fully wrapped (Figure 2a,III) or (2) a single lobe is being wrapped, such that the dumbbell is engulfed up to its waist by the membrane (Figure 2b,VI). The green-blue points in Figure 2a,b represent dumbbells attached almost parallel to the membrane at the beginning of the process (Figure 2I,IV), whereas the yellow-red points represent dumbbells attached roughly perpendicular with respect to the membrane initially (Figure 2II,V). Other starting orientations also lead to either a fully wrapped or a half wrapped dumbbell, but the probability for reaching either state was influenced by the initial position as we will discuss below.

**Figure 2:**
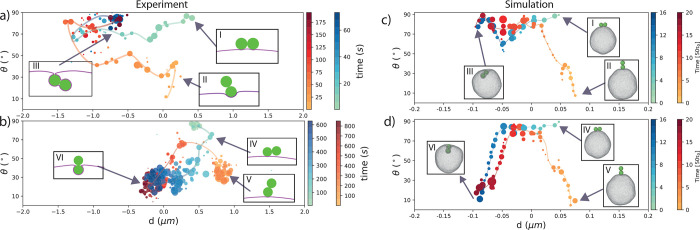
Quantitative wrapping pathway of dumbbell particles by GUVs. Tilt angle θ and distance *d* of the dumbbell from the vesicle surface obtained from (a,b) experiments and (c,d) simulations as a function of time. In all panels, green-blue pathways indicate dumbbells starting from a vertical position with respect to the vesicle surface, and yellow-red pathways indicate dumbbells that initially start almost horizontally with respect to the membrane. Time is indicated by color and specified by color bars for each panel. (a) Experimentally obtained pathways for a dumbbell initially oriented parallel (I) or perpendicular (II) to the membrane surface to a fully wrapped end state (III). Each data point represents an average over 1 s. (b) Experimentally obtained pathways taken by a dumbbell initially oriented parallel (IV) or perpendicular (V) to the membrane surface to the half-wrapped end state (VI). Each data point represents an average over 5 s. (c) Simulations of pathways for a dumbbell initially oriented parallel (I) and perpendicular (II) to the membrane surface to the fully wrapped end state (III). This was the most common stable state with ∼90% of dumbbells reaching this end state. (d) Simulation of pathways for a dumbbell initially oriented parallel (IV) and perpendicular (V) to the membrane surface to the half-wrapped end state (VI). (a–d) Circle size indicates the number of images used for the average and the smooth lines guides to the eye. Simulation time is expressed in τ_0_, the MD unit of time.

If the dumbbell is oriented parallel to the membrane initially (θ ≈ 90°) and proceeds to a fully wrapped state, then it tilts in the first part of the engulfment process to about 60°. Subsequently, its CoM moves inward to almost *d* ≈ 1.5*d*_*s*_ from the undisturbed membrane contour, before returning to a more parallel orientation and an insertion depth about *d* ≈ 0.7*d*_*s*_. This overshooting and recoil is similar to that observed for spheres previously.^8,33^ If the dumbbell initially is roughly perpendicular the membrane, then it first becomes oriented more precisely perpendicular until it is covered halfway (*d* = 0 and θ ≈ 10°) before being wrapped further and finally ending in a more parallel orientation at a similar distance from the undisturbed membrane as the initially parallel dumbbells. Due to the spherical symmetry of the lobes, the point where the membrane peels off from the particle and makes a catenoid-shaped neck with the vesicle is not uniquely determined. Thus, the angle the dumbbell makes with the membrane after having been fully wrapped can vary as it is determined by random processes such as the inhomogeneity of the NeutrAvidin coating and thermal fluctuations.

For final states where only one lobe is being wrapped, an initially perpendicular dumbbell first reorients more parallel before becoming engulfed up to its waist while becoming perpendicular again. An initially parallel dumbbell proceeds to reorient perpendicular while being engulfed; see Figure 2b and Movie S2. The gap in the yellow-red trace at θ ≈ 55° and *d* = 0.5 μm was caused by the dumbbell going through an orientation that was filtered out for accuracy as described above.

To obtain more quantitative results for the dynamics of the system we carried out coarse-grained (CG) molecular dynamics (MD) simulations of anisotropic dumbbell particles being wrapped by a membrane. Besides the advantage of easily measuring dynamic properties, in these simulations we are also able to control the size of the vesicle and dumbbell, the membrane tension and the interaction strength between dumbbell and membrane and thus probe a wider parameter space than is available to experiments.

The membrane is modeled using a one particle thick fluid surface developed by Yuan et al.^34^ which reproduces the mechanical properties associated with biological membranes.^35^ Using this model, we simulate spherical membrane vesicles and change the membrane tension by the addition of small solute particles on the inside and outside of the vesicle.^36^ The solute particles only interact via volume exclusion and produce a pressure force when the inside and outside concentrations are different. The dumbbell colloid is then placed on the membrane in either a vertical or horizontal initial condition and due to the attractive interaction between the membrane beads and the dumbbell, the dumbbell is slowly wrapped and engulfed by the vesicle. Details can be found in the Supporting Information.

The results obtained from simulations show qualitatively similar behavior as in the experiments, see Figure 2. Again, both final states, i.e., (i) one lobe attached and (ii) fully engulfed, could be reached from any initial position, and the pathway they took was influenced by the initial orientation. Interestingly, our simulations suggest that the initial position strongly influences the first part of the wrapping process and to a lesser degree the second half, which is observed to be similar for both extreme initial orientations. The observation that the wrapping pathways from different initial positions can result in the same final position shows that there is an energy minimum for the GUV-dumbbell system independent of the initial position of the dumbbell. In all observed pathways toward the fully wrapped state, the dumbbell particle tilts during the engulfment suggesting that this requires less bending energy.

A similar reorientation upon wrapping was observed for linear aggregate of particles^37^ and elongated ellipsoids.^21,25−27^ Ellipsoids have been found to become first adhered by the side, before rotating to the tip upon being wrapped by the membrane.^25^ For sphero-cylindrical particles that were initially touching with their tip, a rotation-mediated wrapping was also seen,^17,23^ which can rotate the particle from a standing to a lying position when the aspect ratio is high. The first point of contact has been predicted to be crucial for the ultimate fate of a nonspherical particle.^26,27^ In contrast, for the dumbbell particles used here rotation is not driven by a variation of particle curvature but primarily by thermal fluctuations and possibly inhomogeneities in the ligand coating density, because of the constant curvature of the constituent spheres of the dumbbells. The only region of curvature variation is the dumbbell neck, which we will show to play a crucial role in the wrapping.

From the many wrapping processes we observed in experiments and simulations, we identified a number of key intermediate states during the engulfment that ultimately determined the final state. A decisive event during the wrapping of the first lobe is whether the second lobe gets bound to the membrane. This is always the case if the particle starts out being perfectly parallel and thus with both lobes attached (Figure 3A3). If the particle initially is attached with a single lobe (Figure 3A1,A2), however, then tilting during the engulfment may attach the second lobe (Figure 3B). In principle, since one lobe is spherical one may expect engulfment to proceed uniformly, not inducing or requiring any tilt. However, any inhomogeneity in the coating density of the ligands on the dumbbells, as well as thermal fluctuations will tilt the particle and may induce contact of the second lobe to the membrane. Since biotin–NeutrAvidin interactions are essentially irreversible at room temperature, attachment of the second lobe always precludes achieving a final state where only one lobe is wrapped. If the second lobe does not attach, then the single-wrapped lobe state is reached (Figure 3D). Otherwise, the dumbbell will wrap both lobes consecutively, either in a symmetric fashion (Figure 3E2) or in an asymmetric way (Figure 3E1), leading to the fully wrapped state. The symmetric wrapping is unstable, and eventually leads to Figure 3F in which both lobes are covered. The angle the dumbbell makes with the membrane after wrapping completed can vary. In this end state, a small neck connected the fully wrapped dumbbell at one lobe with the vesicle; see Figure 3F.

**Figure 3:**
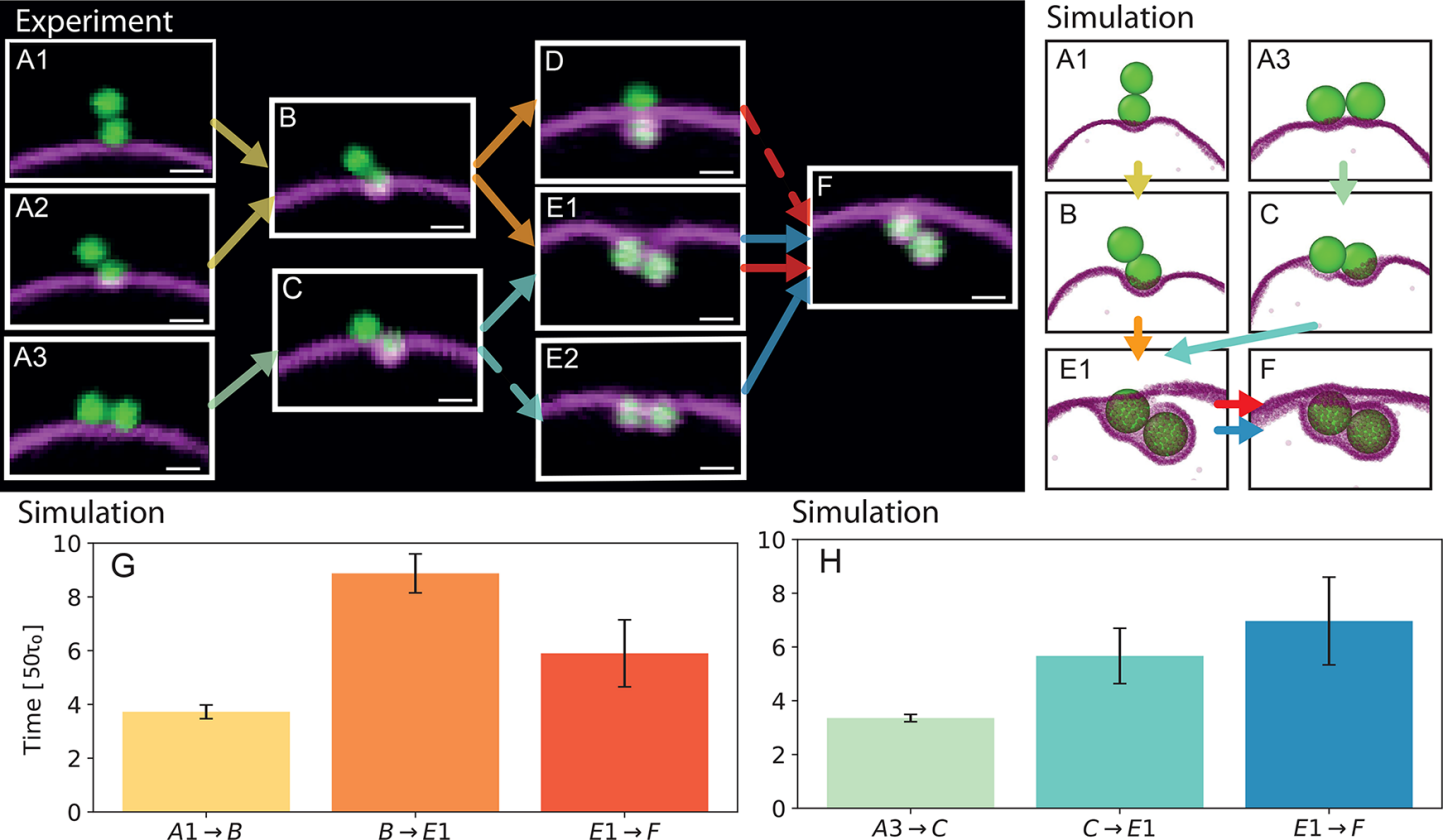
Overview of the observed wrapping pathways. (A1–F) Confocal images of the possible orientation of a dumbbell (All scale bars denote 1 μm). Arrows indicate the directions of the possible wrapping pathways, and dashed arrows illustrate transitions that were rarely observed. (G) Measurements of the time between the states for the vertical dumbbell starting position, given in simulation timesteps. (H) Measurements of the time between the states for the horizontal dumbbell starting position.

To quantify the time evolution, we measured the transition times between the different wrapping states. Membrane tension was found to be crucial for the overall wrapping time, see below. Therefore, simulations were used for quantitative measurements of the transition times and experiments for qualitative comparison. While the initial wrapping of the first lobe in the simulations is fast for the different initial states (see Figure 3G,H), the wrapping slowed down significantly when the membrane was crossing the waist (Figure 3G, B → E1 and H, C → E1). This signifies an energy barrier stemming from the high bending energy required to adapt to the strong variation in curvature of the particle surface. The slowing down at the waist was more significant for the initial condition of a single lobe attached (Figure 3G, B → E1) than the initial condition of both lobes attached (Figure 3H, C → E1). We observed the same qualitative behavior in experiments, both for tense and floppy GUVs, indicating that the bending energy required to continue wrapping largely exceeded the energy gained from adhesion. In experiments, dumbbells typically wrap within 10–200 s after attachment, depending on membrane tension. Particles wrapped with one lobe (as shown in Figure 3D) suddenly transition to the fully engulfed state in less than 10% of these cases within about 10 min. In simulation, the full engulfment took on average (790 ± 30)τ_0_, and the one-lobe state was stable after (980 ± 20)τ_0_ before the membrane broke due to the large binding energy causing the membrane layer to stretch and tear. Therefore, we never observed the transition between states where one lobe is wrapped and where both are fully engulfed within simulations. These observations are in line with ref (13). Indeed, we predict that large membrane fluctuations (of the order 100 nm) would be required to transition from the one lobe to the fully wrapped state if the wrapping was to occur completely symmetrically (details of the full calculation can be found in the SI). The high bending energy costs at the waist and the significantly faster wrapping for tilted dumbbells observed in both simulations and experiments suggest that wrapping a tilted dumbbell is less energetically costly than one that is oriented perpendicular to the membrane.^25^ The strong trapping at the waist also causes single-lobe-wrapped dumbbells to attain their stable insertion depth *d* without overshooting and recoil.

The probability of following a specific pathway and reaching one of the two final states as qualitatively observed in experiments, depended on two factors: the membrane tension of the GUV and the dumbbell’s angle θ_0_ with respect to the membrane’s surface normal during the initial wrapping. The higher the surface tension of the GUV, the more likely it was for the dumbbell to end up in situation Figure 3D. Large fluctuations of the vesicle’s surface enabled the dumbbell to attach to the nonwrapped lobe. The larger the angle θ in situation Figure 3A2, and thus the closer to the membrane it started out at, the more likely it was for the dumbbell to end up in situation Figure 3B and hence Figure 3E1.

The overall time as well as the transition between different stages in the wrapping strongly depended on the membrane tension: Both the initial tension as well as the tension at later times which will increase because of the wrapping, see Figure 4. We experimentally measured the membrane tension from the fluctuation spectrum of the lipid vesicle following ref (38) and plot the time taken to complete wrapping as a function of membrane tension in Figure 4a,b. We observed an increase in overall wrapping time with increasing initial membrane tension in experiments (Figure 4a,b) and simulations (Figure 4c). However, the range of tensions we could replicate in experiments and simulations was quite limited. To be able to fully explore this effect, we extended a previously developed analytical theory describing the time to wrap colloids,^39,40^ which was recently experimentally confirmed,^8^ and adapted it to the shape of a dumbbell (details of the theory can be found in the SI). In doing so we could explore the effect of tension on time to wrap the dumbbell for a range of theoretical parameters. All the parameters used in the theory were taken directly from the experiment, apart from the binding energy per area (*W*) and the microviscosity of the membrane (η_eff_) which are both discussed below.

**Figure 4:**
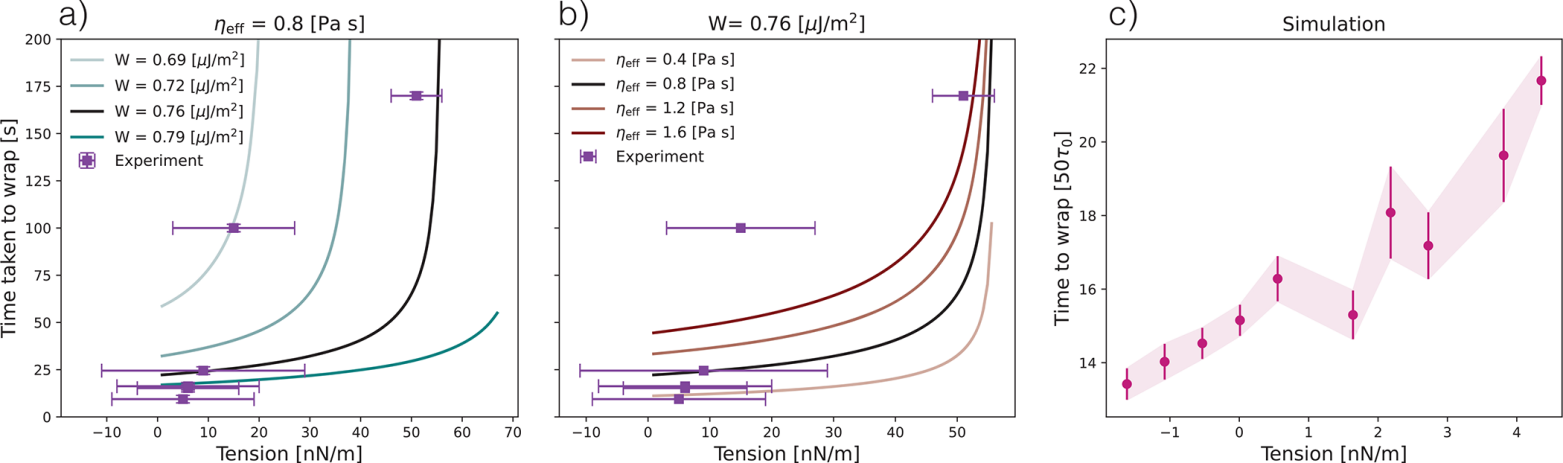
Measurement of the time required to fully wrap a dumbbell-shaped particle as a function of membrane tension. (a) Experimental data (points) and theoretical predictions (lines) for different adhesion energy per unit area in the range of 0.69–0.79 μJ/m^2^ at a fixed membrane viscosity of 0.8 Pa·s. (b) Experimental data (points) and theoretical predictions (lines) for different membrane viscosity in the range of 0.4–1.6 Pa·s at a fixed adhesion energy per unit of area of 0.76 μJ/m^2^. (c) Time to fully wrap the dumbbell-shaped particle in simulations for a range of tensions of <10 nN/m.

For a given adhesion energy, we find that the time taken to fully wrap the dumbbell increases nonlinearly with the tension. With increasing adhesion energy, the wrapping process becomes faster at the same tension, see Figure 4a. The adhesion energies in experiments vary due to the distribution of binding sites between dumbbells^18,31^ which is also reflected in that the experimental data points fall within a range of adhesion energies identified by the theory. We note that only a small percentage of the NeutrAvidin sites that have been added during synthesis contribute to the effective adhesion energy, as was found previously in ref (18). Although fixed in the experiments, varying membrane microviscosity in the theory also changes the time taken to wrap. Membrane microviscosity is a measure of how easily the lipids slide past each other during rearrangement, and a higher microviscosity is linked to a higher frictional force during colloid-membrane wrapping. The comparison between the theoretical and experimental results allows us to estimate the membrane microviscosity, which is experimentally inaccessible. We find that our experimental measurements best fit the theoretical curves for a membrane microviscosity of η_eff_ ≈ 0.8 Pa·s (Figure 4b), about 10 times larger than the lower bound estimated in ref (8). However, the theory in ref (8) consistently overestimated the wrapping speed as compared with experiments on spheres, so it could be that the experiment microviscosity was larger than their theoretically predicted value.

Here we have developed the first model system to quantitatively study ligand–receptor mediated endocytosis of an anisotropic object by making use of GUVs and colloidal dumbbell particles. We followed and quantified their orientation θ and distance *d* with respect to the membrane during wrapping using experiments and molecular dynamics simulations. We found that there are two final states: (1) Only one lobe or (2) both lobes of the dumbbell are fully wrapped by the membrane. The two states can be reached from any initial position except when both lobes were attached initially which necessarily leads to full wrapping of both lobes. However, the initial position influenced the pathway toward the final state. We identified a number of key intermediate states during the wrapping that determine the final state. Wrapping of one lobe was only found for high membrane tensions and if the other lobe did not touch the membrane during engulfment. Using molecular dynamics simulations, we quantified the time required between key intermediate steps, with the slowest step being the crossing of the highly curved neck region of the dumbbell. With simulations, we confirmed the experimentally observed trend of time to wrap increasing for increasing tension, and using analytical theory, we estimated the membrane microviscosity.

Our results contribute to a better understanding of how shape affects endocytosis, nutrition uptake, and bacterial evasion. Our choice of a simple anisotropic object, a dumbbell, enabled a key insight: Highly negatively curved regions may dominate the wrapping and possibly even prevent full engulfment unless active processes are present. This suggests that objects, such as certain viruses such as pox virus^4^ that rely on endocytosis, may profit from having a convex shape. Incorporation of active processes, such as those driven by actin or ESCRT-III polymers, could provide further insights into how the competition between the passive and active processes affects wrapping. In addition, realizing reversible adhesion through a weaker interaction between the membrane and colloids, for example, by depletion interactions^7^ or weaker, multivalent ligand–receptor based bonds,^41^ would complement our current understanding of the wrapping of anisotropic particles.

## Supporting Information Available

Details of the experiments and simulations; experimental materials used; methods employed for membrane preparation, particle functionalization, experimental imaging, and quantification of ligands on dumbbells; details of quantification of dumbbell wrapping and filters applied for the analysis; detail of theory used for time taken to wrap a dumbbell; alternative presentation of Figure 2 (PDF)
Movie S1: Movie showing an experimental measurement of the wrapping process of a dumbbell colloid attached to a GUV which becomes fully wrapped, recorded with a confocal microscope at 59 frames per second and 3× accelerated (MP4)
Movie S2: Movie showing the wrapping process of a dumbbell colloid that becomes stuck in the one lobe-state during the wrapping process, recorded with a confocal microscope at 59 frames per second and displayed in real-time (MP4)
