# Supplementary material for: Wrapping Pathways of Anisotropic Dumbbell Particles by Giant Unilamellar Vesicles
Source: Nano Lett. 2023 May 4;23(10):4267–73. doi: 10.1021/acs.nanolett.3c00375 (PMC10214445; doi:10.1021/acs.nanolett.3c00375)
Supplement: Supplementary file 1 — nl3c00375_si_001.pdf [file nl3c00375_si_001.pdf]

SUPPORTING INFORMATION FOR
Wrapping pathways of anisotropic dumbbell particles by giant unilamellar vesicles

Ali Azadbakht,[†,‖] Billie Meadowcroft,[‡,¶,‖] Thijs Varkevisser,[†,§,‖] Anđela Šarić,[‡] and Daniela J. Kraft[*,†]

†*Soft Matter Physics, Huygens-Kamerlingh Onnes Laboratory, Leiden University, PO Box 9504, 2300 RA Leiden, the Netherlands*

‡*Institute of Science and Technology Austria, 3400 Klosterneuburg, Austria*

¶*Department of Physics and Astronomy, Institute for the Physics of Living Systems, University College London, London WC1E 6BT, United Kingdom*

§*Van der Waals-Zeeman Institute, Institute of Physics, University of Amsterdam, Science Park 904, 1098 XH Amsterdam, Netherlands*

‖*These authors contributed equally to this work.*

E-mail: kraft@physics.leidenuniv.nl

Materials and Methods

Materials

Chloroform ($CHCl_3$) 99.0-99.4%, D-glucose, EDC N-(3-Dimethylaminopropyl)-N'-ethylcarbodiimide hydrochloride (98%), Sulfo-NHS (N-Hydroxysulfosuccinimide sodium salt) were purchased from Sigma-Aldrich. DOPC (Δ 9-cis 1,2-dioleoyl-sn-glycero-3-phosphocholine), DOPE-

rhodamine (1,2-dioleoyl-sn-glycero-3-phosphoethanolamine-N-(lissaminerhodamine B sulfonyl)), DOPE-PEG2000-biotin (1,2-dioleoyl-sn-glycero-3-phosphoethanolamine-N-[biotinyl(polyethylene glycol)-2000]) were purchased from Avanti Polar Lipids. Potassium chloride (KCl) 99+%, and Sodium azide (NaN_3) 99%, extra pure were provided from Acros Organics. Biotin-4-fluorescein ($C_{33}H_{32}N_4O_8S$) from Molecular Probes. mPEG5000-NH_2 Methoxypolyethylene glycol amine M.W. 5000 from Alfa Aesar. NeutrAvidin was bought from Thermo Fisher Scientific. Phosphate buffered saline (PBS) tablets from Merck Millipore. Ethanol (C_2H_5OH) from Honeywell, and water (H_2O) was filtered with a MilliQ MilliPore apparatus (resistivity 18.2 MΩ· cm). All chemicals were used as received.

Membrane preparation

Electroformation was used to prepare GUVs with a diameter between 10 and 100 µm.[1] A lipid mixture containing 97.5 wt% DOPC, 2 wt% DOPE-PEG2000-biotin and 0.5 wt% DOPE-rhodamine was dissolved in chloroform at 1.45 g l^{-1}. Two 6 cm^2 ITO-coated glass slides (15-25 Ω m^{-1}) were spin coated with 10 µL lipid mixture each, using a SCS 6800 spin coater at 2000 rpm. The coated slides were dried in a vacuum chamber for 2 hours, after which they were placed in 1.8 ml of 100 mM glucose solution and connected to a function generator. An alternating current of 1.1 V(rms) at 10 Hz was applied for 3 hours. The GUVs were stored in a 2 ml Eppendorf tube at room temperature for a maximum of 4 days.

Particle Functionalization

Carboxylic acid functionalized polystyrene spheres with a diameter of 0.98 ± 0.03 µm were prepared using a surfactant-free dispersion polymerization protocol.[2] These spheres were coated with NeutrAvidin and mPEG5000-NH_2, based on a modified version of the protocol described in.[3] The modification enabled the particles to cluster and form dumbbells during the coating process. All reactions were performed at 4 °C. First, particles with concentration

of 2%w/w were activated using 8 mM EDC and 2.5 mM Sulfo-NHS. After 30 minutes of vortexing, 1 µmol NeutrAvidin dissolved in 50 µL water was added to 750 µL of activated particles. After 2 hours of vigorous mixing with a vortex mixer, 200 µL water containing 4.0 mg dissolved mPEG5000-NH_2 was added and the entire solution was mixed using an orbital rotator at 30 RPM for 40 hours while still at 4 °C. Last, the coated particles were washed three times with water and 3 mM sodium azide was added to prevent bacterial growth.

The particles were coated with polyethylene glycol (PEG) to prevent them from clustering after synthesis. The PEG functionality of the particles was tested by placing the particles in a 1 M KCl solution for one hour. After one hour, no significant clustering of the particles was observed, concluding that the particles were successfully coated with PEG.

Imaging and optical tweezers setup

Imaging was performed on polyacrylamide-coated coverslips, which were prepared following a protocol based on.[4] The coverslips were washed thoroughly with water and PBS before use. Besides, GUVs were washed with PBS to remove undesired lipid structures. Samples were prepared by placing 400 µL of this mixture on a polyacrylamide-coated coverslip and kept open to evaporation to gradually increase osmolarity. A Nikon Eclipse Ti microscope with an A1R confocal scanning head and a 60× (NA = 1.2) water immersion objective were used to acquire all images. The sample was placed on a MCL Nano-Drive piezo to acquire high speed z-stack images. The attachment of a dumbbell to a GUV was established by bringing the particle within close proximity of the GUV using an optical tweezers setup. The optical trap is a home-built setup consisting of a highly focused 1064nm laser beam of Nd:YAG Opus purchased from LaserQUANTUM. The laser beam enters through the rear aperture of the microscope and is reflected by a dichroic mirror onto the rear pupil of the objective. While control of the particle position is very high, the orientational control is only approximate. Dumbbell colloids orient themselves in the trap such that their long axis lies perpendicular to the imaging plane. Approaching a GUV from the top, the particle typically

adheres with only one lobe, whereas approaching it at the equator the particle is more likely to adhere with either both lobes or with a higher value of θ.

Analysis

All images were analysed with python. A tracking toolkit called Trackpy[5] and an extension based on this toolkit called Clustertracking[6] were used to determine the two dimensional coordinates of both lobes of the dumbbell particles. The two dimensional GUV contour was determined using a package called circletracking.[7]

During the data acquisition, z-stack image sequences were captured for all examined GUVs. A z-stack image sequence consisted of multiple images recorded at different focal heights. By ascending the z-coordinate in small steps (typically 300 nm) from the bottom to the top of a GUV, the complete vesicle was imaged. The radius of the GUV was then determined from the equator image of the z-stack. All radii were determined by averaging over multiple equator images from different z-stacks.

Quantification of specific binding affinity

The NeutrAvidin functionality was confirmed using the specific binding affinity to biotin, by showing that the particles were wrapped by biotin-functionalised GUVs. The average amount of binding sites per particle was quantified by titration analysis, using the same method discussed in.[3]

First, NeutrAvidin-functionalised particles were dispersed with a concentration of 0.05% w/w in PBS and different amounts of biotin-4-fluorescein were added to the particles. The mixture was then incubated for 20 minutes at 55 °C. After incubating, the particles were sedimented by centrifuging and the supernatant was collected. The fluorescence of the diluted supernatant was subsequently measured using fluorescence spectrophotometry. The added amounts of biotin-4-fluorescein were selected such that some amounts were completely absorbed by the NeutrAvidin-functionalised particles, while others remained partly in the

supernatant. A horizontal baseline and a linear function were fitted, respectively, to the two sections. The amount of absorbed biotin, and thus the binding affinity of the particles was determined from the intersection point of both fits. The absorbed biotin was 23.5 pmol mg^{-1} which implied an average amount of 7.3×10^3 binding sites per spherical particle, and twice that amount for dumbbells. Assuming 17 $k_B T$ per avidin-biotin bond,[8] the adhesion energy per unit area was estimated to average 156 $\mu J/m^2$, which is well above the critical value for wrapping one sphere. It is worth mentioning in earlier work on the same system,[9] it was found that there is an order of magnitude spread in the Neutravidin coating density of particles. Moreover, after applying the Neutravidin coating, we subsequently stabilize the particles against aggregation with an additional layer of PEG polymers discussed in Particle Functionalization. These polymers are likely to partially block access and thus adhesion of biotinylated lipids to the Neutravidin. The titration assay of the Neutravidin density with fluorophores functionalized with biotin are more likely to still bind due to their much smaller size and greater freedom to move compared to the biotinylated lipids in the membrane, which leads to an overestimation of the accessible Neutravidin on the particle surface. This overestimation may further be enhanced by the fact that at least three out of the four Neutravidin binding pockets will be oriented such that access to them is obstructed by the polymer coating. The earlier study showed that only 2% of the linkers are able to effectively adhere to the ligands in such an experimental system.[9]

The results of the fluorescence measurements and the two fits are illustrated in figure S1. The baseline has a non-zero value as the biotin-4-fluorescein had the same emission wavelength as the fluorescent dye included in the particles, and some of the particles remained within the supernatant after centrifuging.

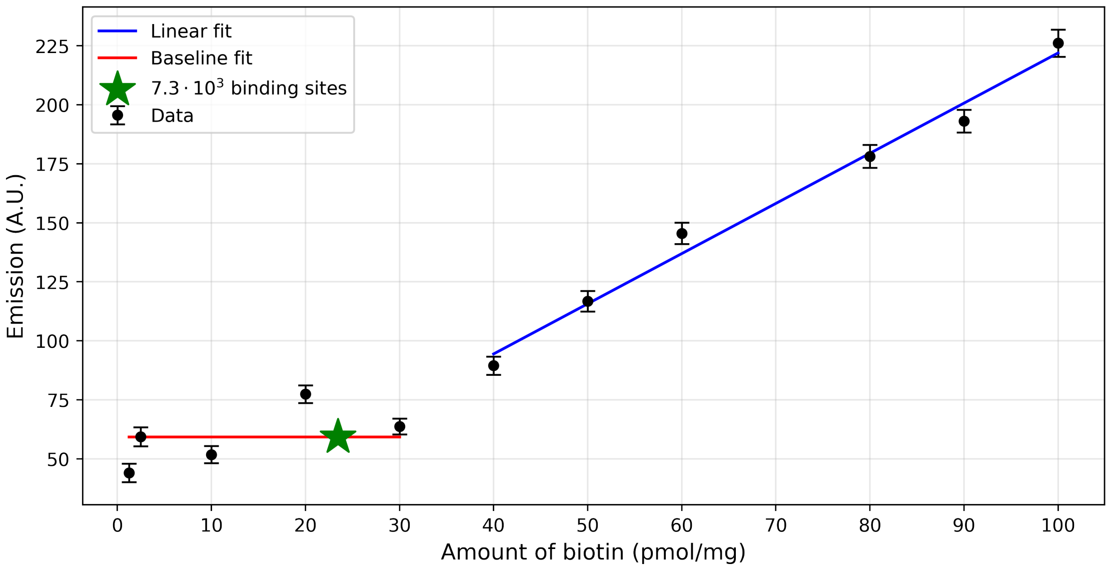

Figure S1: Emission intensity of the supernatant of biotin-4-fluorescein (b4f) incubated, NeutrAvidin-functionalised particles for different amounts of b4f. The baseline is fitted for the amounts of b4f that were completely adsorbed to the particles. The linear function is fitted to amounts where some b4f remained in the supernatant after centrifugation. The green star indicates the intersection of the two fits, and corresponds to 7.3×10^3 binding sites per spherical particle.

Quantification of dumbbell wrapping

We quantified the wrapping process by measuring the dumbbell's angle θ with respect to the membrane's surface normal and the distance d between the dumbbells center of mass (CoM) and the membrane. To do so, we first measured the GUV radius R and its center by fitting a 3D confocal Z-stack with a sphere using the Python package circletracking.[7] Subsequently, we tracked the position of the two lobes of the dumbbell using the Python package clustertracking[6] for each time frame and from this calculated the position of the dumbbell's center of mass (CoM) in time. Measuring the cross-section radius r of the GUV in each frame, see Fig. 1f, we determined the distance of the dumbbell from the equator of the vesicle in z, through $z = \sqrt{R^2 - r^2}$, since measuring of z allowed a 3D tracking of all parameters. In addition, because the quality of the contours decreases far from the equator, the particles were tracked only when -0.8R<z<0.8R. Then, given R and a dumbbell CoM distance to the centre of this sphere q, the dumbbell CoM distance d to the vesicle can be

defined as

$$\mathrm{d} = q - R \tag{1}$$

Knowing the absolute position of the dumbbell and vesicle in three dimensions, we can calculate the distance of the dumbbell's CoM from the undistorted membrane contour, d, as well as the angle θ between the major axis of the dumbbell and surface normal of the GUV, which are depicted in Fig. 1d. We approximated the position of the membrane in the vicinity of the dumbbell-induced deformation by fitting a circle to the membrane. We note that we only analyzed frames where the dumbbell was approximately in the confocal plane. Since the fluorescence intensity decreases as an object moves out of focus, we selected frames for analysis where the intensity of both spheres were not lower than 75% of the maximum intensity of each lobe and differed by no more than 20% from their average intensity. In this way, the precision of θ was increased, with a maximum error of 19°. For details, see below Experimental image filtering.

Due to the indentation of the membrane induced by the dumbbell, the membrane's contour deviated from a perfect circle. Therefore, we locally fitted the membrane contour in the vicinity of the particle to obtain a more accurate results of d. To do so, we disregarded the highly deformed membrane contour in the direct vicinity of the particle up to 3.5μm around the CoM, see Figure S2. The negative bias induced by the global circle fit was prevented by ignoring this contour. Additionally, a contour of azimuthal angle more than $\pm 36°$ away from the particle was also disregarded in the local circle fit, such that the circle approximated the contour in the vicinity of the dumbbell as well as possible, indicated with yellow dots in fig. S2.

Figure S2 shows both the globally and the locally fitted circle for a tense and a floppy vesicle. The yellow dots illustrate the selected contour to which the local circle fit was performed. The figure also shows that in the case of a tense vesicle without any wrapped

particles (the depicted dumbbell is attached only), the global and local circle fits well. However, the locally fitted circle approximated the contour around the particle better than the global circle fit for the floppy vesicle with a partly wrapped dumbbell. For this reason, the locally fitted circle was used to calculate the dumbbell's center of mass distance d to the GUV's surface.

Experimental image filtering

GUV radius filtering To prevent substantial errors in the GUV contour determination due to noise, only frames around the equator were used. The selection of suitable frames was made by fitting the vesicle contour for all frames with a circle and comparing it to the radius R of the GUV obtained from fitting a sphere to a z-stack. Images where the radius of the circle was smaller than $0.8R$ were not used in the analysis. The boundary value of 80% was determined by manually assessing the quality of multiple GUV contours at various distances from the equator. A further increase in selectivity to images whose radius falls within 90% of the vesicle radius is shown in Fig.S3b but did not further improve reduction of noise. Thus, we selected images where the radius of the vesicle fell within $0.8R$ for the whole study.

Filtering related to dumbbell intensity To ensure high precision of the quantitative analysis of the dumbbell wrapping process, we proceeded as follows: we used the particle tracking algorithm to determine the coordinates as well as to calculate the intensities of both lobes. The coordinates were determined by fitting a Gaussian to the intensity profiles, and the sum of all the pixel intensities within a profile was reported as the lobe intensity. We then applied two filtering steps to ensure that the dumbbell lobes were at the same height and located in the focal plane.

To improve the accuracy of the z-position, we selected frames where both dumbbell lobes were at the same height and within error in the optical focal plane of the objective lens. This

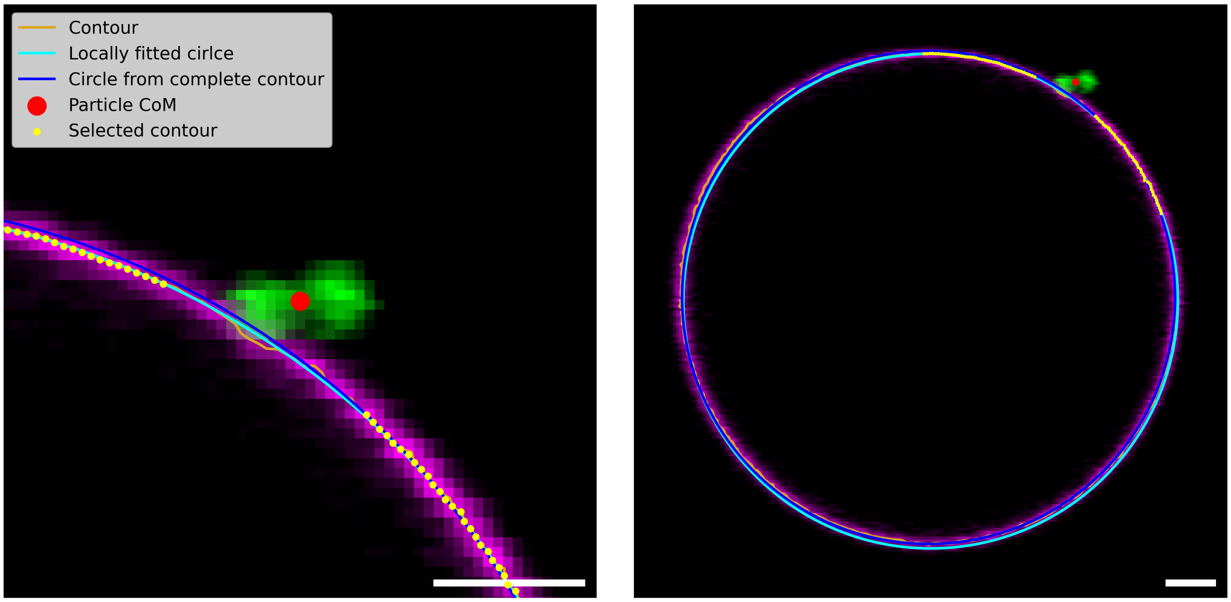

(a) Tense vesicle, close-up of (B)

(b) Tense vesicle

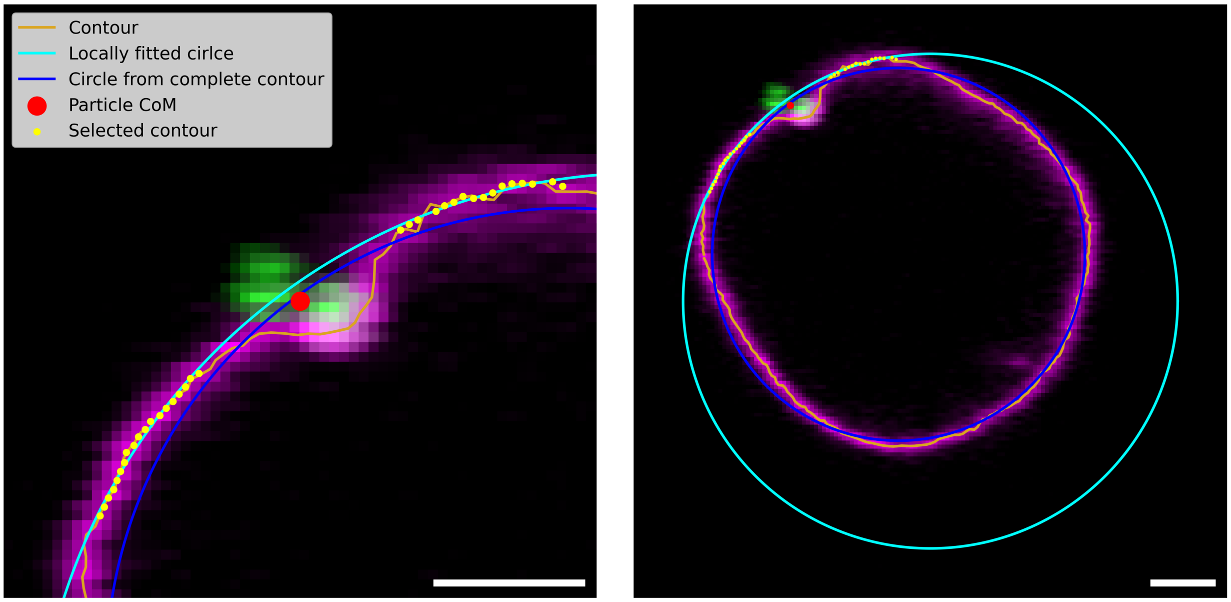

(c) Floppy vesicle, close-up of (D)

(d) Floppy vesicle

Figure S2: Confocal images illustrating the global and local circle fit for a (A,B) tense vesicle and a (C,D) floppy vesicle. The dumbbell particles are depicted in green and the vesicle in magenta. For the tense GUV without any wrapped particles, the locally fitted circle and the circle fitted to the complete contour both match the contour around the particle very well. For the floppy vesicle with the (partly) wrapped particle, the locally fitted circle matched the contour around the particle better than the global circle fit. The contour used to locally fit the circle is highlighted with yellow dots. All scale bars denote 2 µm.

was accomplished by selecting frames where the intensity of the spheres was within 75% of the maximum lobe intensity observed in a z-stack scan of the GUV-dumbbell system. A further increase in the filter value to 85% of the maximum lobe intensity does not further reduce the noise but only removes additional data points, see Figure S3 c.

To make sure that the two lobes of the dumbbells were both located in the focal plane, we introduced a relative intensity filter. To do so, we measured the mean normalized intensity of the two spheres in every frame and removed those frames where a normalized lobe intensity deviated more than 20% from the mean intensity. Reducing the cutoff value to 10% of the mean normalized intensity does not lower the noise in θ as shown in Figure S3 d. Therefore, we chose a value of 20%.

Analysis of measurement error in θ We estimated the error in θ by measuring the error in the relative height of the two dumbbell lobes. To do so, we first measured the average lobe intensity as a function of z by scanning a single sphere in steps of 1 nm with confocal microscopy. 12 separate z-stack image sequences were used to determine the average intensity as a function of the z height relative to the sphere's maximum intensity value, see Figure S4aa. This maximum value was determined by fitting a parabola to the intensity. We set the z coordinate corresponding to the maximum of the parabola as the value the sphere would have if it is in the focal plane during a measurement of the wrapping process, i.e. $z = 0$. The standard deviation from these 12 measurements relative to the average intensity is depicted in figure S4bb.

The standard deviation (stdev) in the average intensity implies that the z-position of the two lobes of the dumbbells can only be determined up to that value through this method. For $z = 0$, the value of the normalised standard deviation stdev$(z = 0) = 0.05$ corresponds to an error in the z-coordinate of ± 159 nm. Such a deviation in the z-position corresponds to a maximum measurement uncertainty in the angle $\Delta\theta = 19°$ between the dumbbell long axis and the focal plane when the dumbbell lies approximately in the plane, see Figure S5.

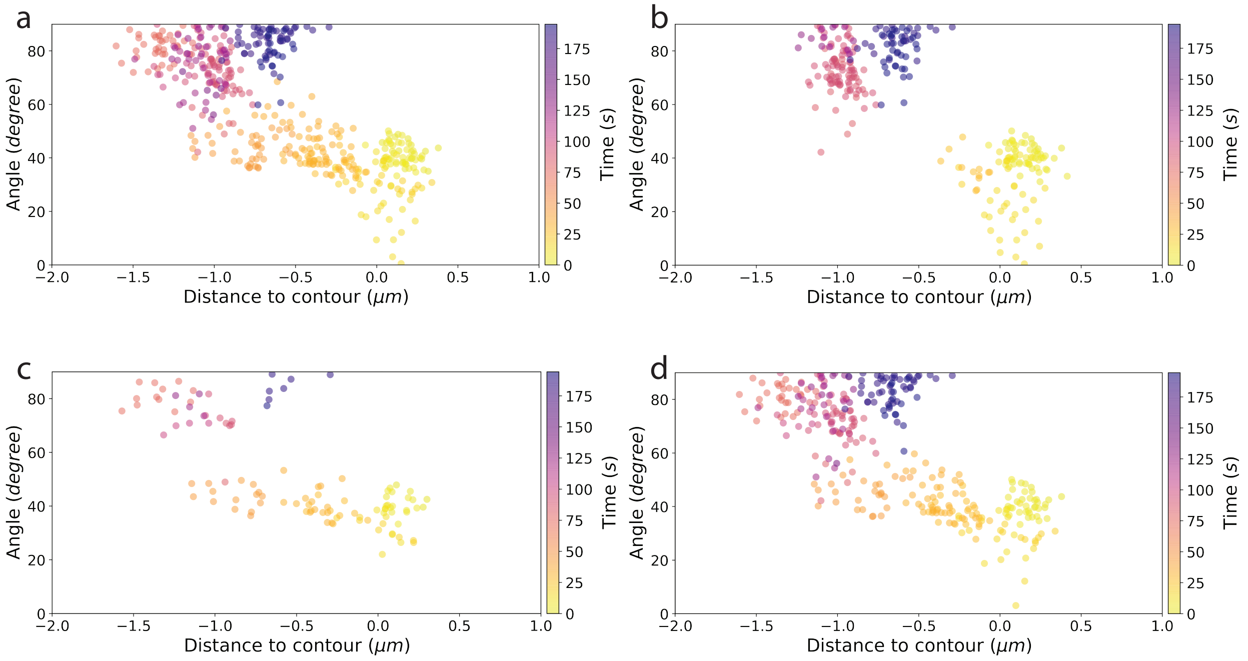

Figure S3: Quantitative wrapping pathways as presented in Figure 2a for different filter settings. Depicted are θ and d as a function of time, which is indicated by color coding. . (**a**): The pathways for the default filter values in which the radius of the circle was not allowed to be smaller than 80% of the actual GUV radius, the sphere intensity had to be higher than 75% of the maximum intensity of each lobe, and the relative dumbbell lobe intensity had to be higher than 80% of the mean intensity value. (**b-d**): Pathways for different settings of the filter values. **b** Frames with GUV crossesctions smaller than $0.9R$ were filtered out. **c** Frames where lobes with an intensity that was below 85% of the maximum lobe intensity were filtered out. **d** Frames where the relative intensity deviation of each lobe was less than 90% of the mean normalized intensity were filtered out. None of the increased filter boundary values resulted in a significantly reduced fluctuations of the angle-distance data.

Simulations

Simulation details

We ran molecular dynamics simulations using LAMMPS[10] and explored a membrane vesicle wrapping a dumbbell-shaped colloid. The membrane was modelled using a 1-bead thick membrane model developed by Yuan et al[11] which models an implicit solvent with a single anisotropic pair interaction potential. The parameters for the membrane were as follows: $\sigma = 1$, $r_{\text{cut}} = 2.6$, $r_{\text{min}} = 1.12$, $\mu = 3$, $\zeta = 4$, $\epsilon = 4.43$, following the notation in the original paper. The membrane vesicle is made of $N = 9722$ beads. The dumbbell was a single rigid

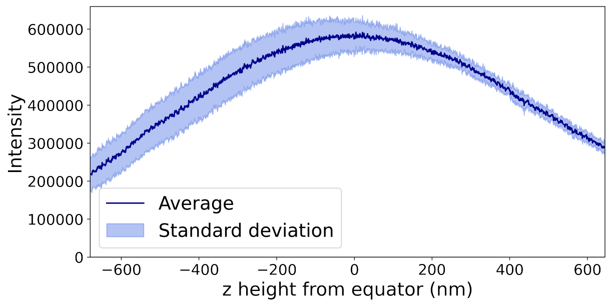

(a) Single particle intensity

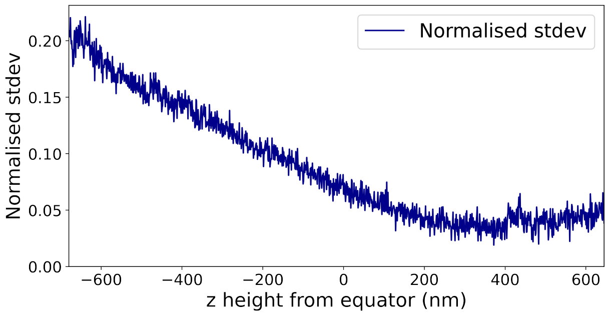

(b) Average-normalised standard deviation

Figure S4: The average intensity and standard deviation from 12 z-stack image sequences of a single fluorescent spherical particle. (**A**): The average intensity and standard deviation as a function of the z height from the particle equator. $z = 0$ was determined by fitting a parabola to the average intensity and using the maximum value. (**B**): The standard deviation normalized by the average intensity as a function of the z height.

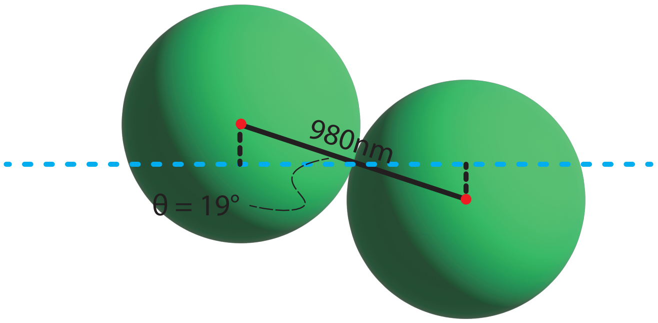

Figure S5: Illustration of error in the angle θ of a dumbbell with the focal plane (dashed blue line) given a standard deviation of 159 nm for the z-coordinate.

body consisting of two spheres, each with a diameter $\sigma_{\text{dumbb}} = 7.5\sigma$. We convert this to real units in Figure 2 using $\sigma = 10$ nm. The mass of each dumbbell sphere was 30× larger than one membrane bead. The dumbbell interacted with the membrane at a distance r_{ij} via a cut-and-shifted Lennard-Jones potential: $E_{ij} = 4\epsilon((\frac{\sigma}{r_{ij}})^{12} - (\frac{\sigma}{r_{ij}})^6) - E_c$, $r_{ij} < r_c$ where $E_c = 4\epsilon((\frac{\sigma}{r_c})^{12} - (\frac{\sigma}{r_c})^6)$. The cutoff was chosen to be $0.65(\sigma + \sigma_{dumbb})$. To qualitatively match the experimental wrapping pathways we found the optimal interaction strength to be $\epsilon = 8k_{\text{B}}T$. The simulations typically ran for $200000\Delta\tau$ where $\Delta\tau = 0.01\tau_0$ (τ_0 is the MD unit of time). The simulations were performed in the canonical ensemble with periodic boundary conditions by coupling an NVE setup and a Langevin thermostat using a friction coefficient $\gamma = \frac{m}{\tau_0} = 1$. The simulation box is a cube with a fixed edge length of $L_{\text{box}} = 92\sigma$.

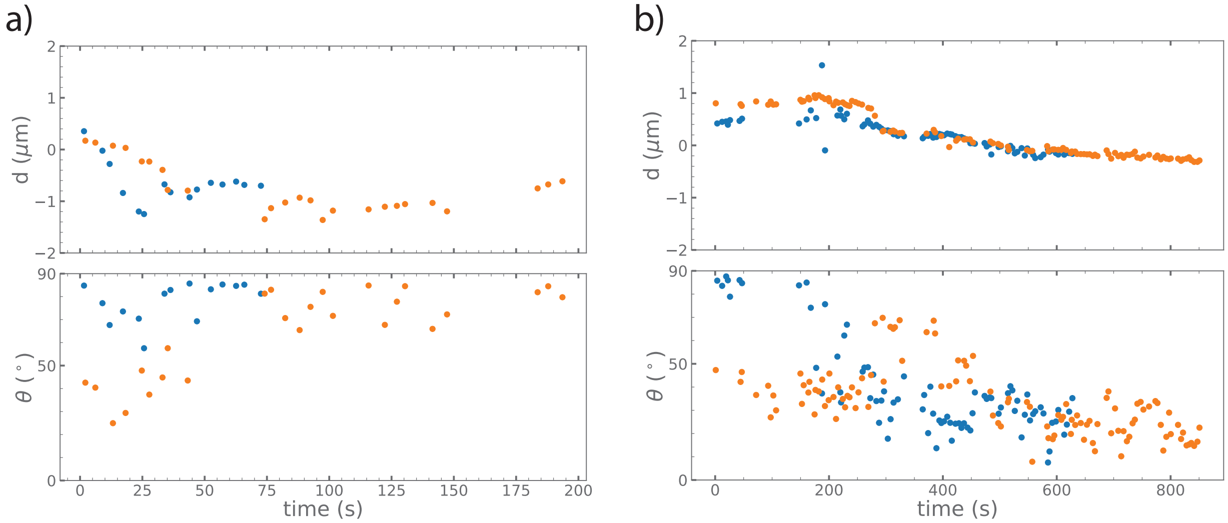

Figure S6: **Alternative presentation of the data shown in Figure 2 a) and b) of the main text.** Distance d of the dumbbell from the vesicle surface and tilt angle θ as a function of time obtained from experiments. The orange data points are initially perpendicular to the vesicle, and the blue data points are oriented parallel to the membrane. a) Experimentally obtained pathways for a dumbbell initially oriented parallel (blue) or perpendicular (orange) to the membrane surface to a fully wrapped end state. Each data point represents an average over 1s. b) Experimentally obtained pathways taken by a dumbbell initially oriented parallel (blue) or perpendicular (orange) to the membrane surface to the half-wrapped end state. Each data point represents an average over 5s.

For the quantification of the time taken between states during the wrapping pathway (Fig.3), we defined the states when the dumbbell reached particular thresholds of d and θ, the values of which we outline here. For the pathway of dumbbells starting in the vertical position, the initial state change is that from vertical (state A1, $\theta = 0°$) to tilted (state B, $\theta = 40°$). The dumbbell then gets more tilted and sinks into the membrane (state E1, $\theta = 65°$, $d = -6\sigma$) before sinking to its final position (state F, $d = -8.5\sigma$). For the pathway of dumbbells starting in the horizontal position the first state change is that of the dumbbell sinking from sitting above the vesicle contour (state A3, $d = 5\sigma$) to be in line with the vesicle contour (state C, $d = 0°$). The dumbbell then sinks more and tilts (state E1, $\theta = 65°$, $d = -6\sigma$) before reaching its final position (state F, $d = -8.5\sigma$).

In Figure 4 c) we measure the time to wrap the dumbbell for different membrane tensions. The different tensions are realised by placing small solute beads with different concentrations in and outside the membrane vesicle. These particles interact only via volume exclusion and thereby behave as a gas and have the sole effect of creating a pressure difference between the in and outside leaflet of the membrane. The pressure difference can be calculated using $\Delta\Pi = \mathrm{RT}(\rho_{\mathrm{in}} - \rho_{\mathrm{out}})$ where $\Delta\Pi$, RT and $\rho_{\mathrm{in/out}}$ are the pressure difference, the ideal gas constant multiplied by temperature and the concentration of particles in/out, respectively. We then use Laplace's law to relate this pressure difference to tension, namely Tension = $\frac{\Delta\Pi r_{\mathrm{ves}}}{4}$ where r_{ves} is the radius of the membrane vesicle.

Effect of membrane tension on time taken to fully wrap

Summary of theory for time taken to wrap the dumbbell

Previous works have developed theory to estimate the time it takes membranes to adhere and fully wrap colloids.[12] Recently, a thorough study calculated the time to wrap a spherical particle including comparison to experiments, estimates of experimentally inaccessible parameters and an in-depth discussion about the relevance of the theory in different regimes.[13] Another theoretical work looked at the time to wrap non-spherical objects including cylin-

ders[14] and further addressed stochastic effects within this model.[15] Here, we employ the same theoretical framework as those in the above published works and apply it to a dumbbell-shape colloid. The theory calculates the uptake force, which is a function of the bending energy and stretching energy of the membrane as well the binding energy of the membrane and colloid. This uptake force is then equated to an effective friction force which acts at the contact line between the colloid and the adhering membrane. Experiment has shown that this contact line moves approximately at a linear speed so that the friction force has an effective constant viscosity, η_{eff}. In general, the time taken to wrap depends on the geometry and size of the particle, the adhesion energy per area, W, the bending rigidity of the membrane, κ, the tension of the membrane, σ, and the micro-viscosity of the membrane, η_{eff}. The non-adhered membrane bending is ignored, as it has been shown that it contributes to up to ~ 20 % of the total membrane bending.[14]

We take the radius of each sphere of the dumbbell ($R = 0.5\mu$m) and tensions (0-50 nN/m) directly from experimental measurement. We assume the bending rigidity is similar to most membranes at $20k_{\text{B}}T$. In the experiment, the tension was reduced via osmosis until the membrane was able to wrap and engulf the particles. Therefore we assume that the particle-membrane system is near the critical adhesion energy needed for wrapping. In this system the critical adhesion energy per area for particle engulfment is $w_c \sim \frac{2\kappa}{R^2} + \sigma = (0.65 - 0.7)\mu\text{J/m}^2$ for the experimental tension range. We therefore vary the adhesion energy per area near to and slightly above this value. The final parameter needed to calculate the time to wrap is the membrane microviscosity. This value represents how easily lipids rearrange near the contact line between colloid and membrane. The effective viscosity is difficult to measure experimentally and has been previously estimated for only a handful of membranes.[13,16,17] Varying the value of the microviscosity we find that the theory best fits the experimental measurements for values of η_{eff} ~0.8 Pa s which is 10 times larger than that estimated in,[13] ≈ 0.08 Pa s.

Calculation of time taken to wrap the dumbbell

We based our calculations on a theory previously developed in[12] and extended in.[15] Below is a summary of the ingredients and assumptions of this analytical theory and an outline of how we extended the theory for use with a dumbbell shape.

The main assumption in the theory is that there is a thermodynamic driving force, calculated from the derivative of the total energy of the system with respect to an appropriate line element, and that this can be equated to a frictional force. The frictional force can be calculated when the contact line between the colloid and membrane is moving at constant speed. In this case there is a microviscosity that linearly relates the speed of the contact line with the force exerted on it.

We assume that the membrane in the vicinity of the dumbbell is locally flat with an intrinsic mean curvature of zero. The total energy of the system can be written as follows:

$$E_{\text{tot}} = -\int_{A_d} W \mathrm{d}A + \int_{A_d} 2\kappa H^2 \mathrm{d}A + \sigma \Delta A \tag{2}$$

Where the first term is the colloid-membrane adhesion energy, W is the adhesion energy per area and A_d is the colloid-membrane attached area. The second term is the membrane bending where κ is the membrane bending rigidity and H is the membrane mean curvature. The third term is the membrane stretching term where σ is the membrane tension and ΔA is the change in area from the flat membrane state.

For a given choice of parametrisation, which is dependent on the specific geometry of the system, we define the contact line between colloid and membrane (the line of the front of membrane attachment), with length l_{cl}, as moving in the direction of the line segment s. The frictional force can therefore be written as:

$$F_{\text{fric}} = \eta_{\text{eff}} l_{\text{cl}} \dot{s} \tag{3}$$

And we relate the total energy and frictional force via:

$$F_{\mathrm{fric}} = -\frac{\mathrm{d}E_{\mathrm{tot}}}{\mathrm{d}s} \tag{4}$$

To solve these equations for the shape of a dumbbell, we separate the shape into two spheres with a small cylindrical neck in between, as shown in figure S7b. We then parameterise the above equations using θ, as shown in S7a, for the spherical shapes and z, the z-axis of the cylinder, for the cylindrical neck. We assume the membrane wraps these shapes consecutively, and therefore we can solve the equation for each shape with the appropriate initial and final positions of the contact line, and add the resulting times. For example, the equation for the cylinder will read:

$$\begin{aligned} \eta_{\mathrm{eff}} l_{\mathrm{cl}} \dot{z} &= -\frac{\mathrm{d}E_{\mathrm{tot}}}{\mathrm{d}z} \\ &= 2\pi R_{\mathrm{cyl}}(W - \sigma - \frac{\kappa}{2R_{\mathrm{cyl}}^2}) \end{aligned} \tag{5}$$

The contact line in this case is $l_{\mathrm{cl}} = 2\pi R_{\mathrm{cyl}}$, so that this equation simplifies to $\dot{z} = \frac{w-\sigma}{\eta_{\mathrm{eff}}} - \frac{\kappa}{2\eta_{\mathrm{eff}} R_{\mathrm{cyl}2}}$ and the time is calculated from $T = \int_0^{h_{\mathrm{cyl}}} (1/\dot{z})\mathrm{d}z$. We can write the total time to wrap as follows:

$$T_{\mathrm{wrap}} = \int_0^{\frac{\pi}{2}+\arccos(\frac{R_{\mathrm{cyl}}}{R_{\mathrm{sph}}})} \frac{1}{\nu_{\mathrm{up}} - \nu_\sigma(1-\cos\theta)}\mathrm{d}\theta + \int_0^{h_{\mathrm{cyl}}} \frac{1}{\nu_{\mathrm{up}}^{\mathrm{cyl}}}\mathrm{d}z + \int_{\frac{\pi}{2}-\arccos(\frac{R_{\mathrm{cyl}}}{R_{\mathrm{sph}}})}^{\pi} \frac{1}{\nu_{\mathrm{up}} - \nu_\sigma(1-\cos\theta)}\mathrm{d}\theta \tag{6}$$

Where, as defined in,[15] $\nu_{\mathrm{up}} = \frac{w}{R_{\mathrm{sph}}\eta_{\mathrm{eff}}} - \frac{2\kappa}{R_{\mathrm{sph}}^3\eta_{\mathrm{eff}}}$, $\nu_\sigma = \frac{\sigma}{R_{\mathrm{sph}}\eta_{\mathrm{eff}}}$ and $\nu_{\mathrm{up}}^{\mathrm{cyl}} = \frac{w-\sigma}{\eta_{\mathrm{eff}}} - \frac{\kappa}{2\eta_{\mathrm{eff}} R_{\mathrm{cyl}2}}$.

This can be solved analytically which gives rise to the plots in figure 4. A list of the parameters used for the theory can be found in table S1. The solutions reflect that near to the critical adhesion energy the time to wrap diverges and therefore small changes in W or σ will give rise to large changes in the time to wrap. This can be seen in S8 which shows the

time to wrap near the critical regime for different tensions, and far from the critical regime.

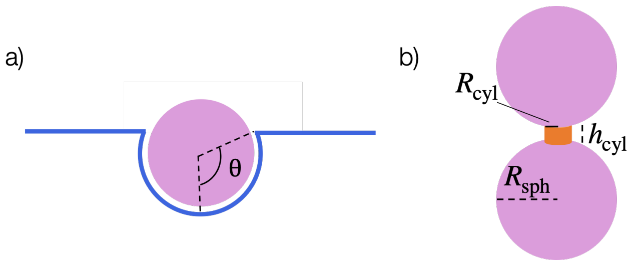

Figure S7: **Shapes for the theoretical time to wrap calculation.** a) For the spherical geometry, we define θ from 0, when the sphere is only just in contact with the flat membrane, to π when the sphere is fully engulfed by the membrane. b) For the dumbbell geometry there are three key geometric parameters: the radius of the sphere (R_{sph}), the radius of the cylinder between the two spheres (R_{cyl}), and the height of this cylinder (h_{cyl}).

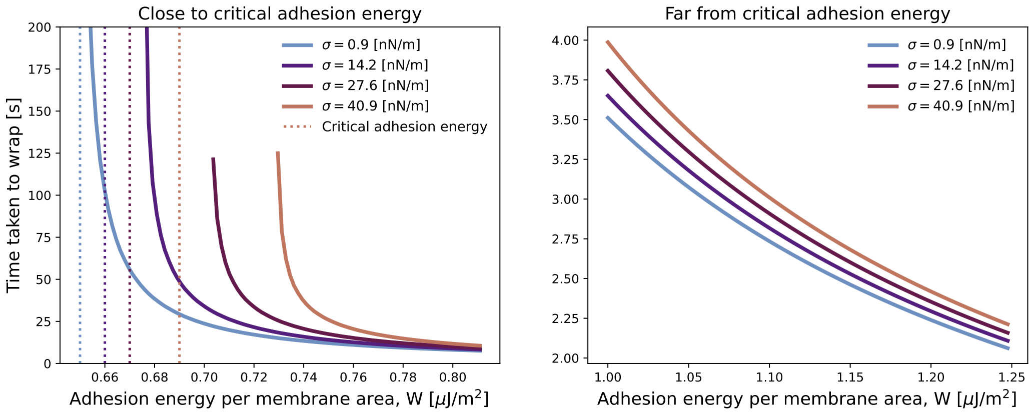

Figure S8: **Solutions to Equation (6) in two regimes**. Time to wrap the dumbbell close to the critical adhesion energy for wrapping (left) and far from the critical adhesion energy (right). The time to wrap diverges at the critical adhesion energy, W_{c}, which is different for different tensions.

Estimating the energy barrier at the dumbbell neck

Our experiments and MD simulations both show a 1-lobe wrapped state that is stable for some time. This suggests that there is an energy barrier to reach the fully wrapped global energy minimum from this local minimum. Here, we use the Helfrich model for membrane bending to estimate an upper bound for this barrier. For simplicity we neglect surface

Table S1: Theory and Simulation parameters

**Parameter**	**Simulation value**	**Theory value**
Membrane rigidity	$17k_\mathrm{B}T$	κ=$20k_\mathrm{B}T$
R_sph	$3.75\,\mathrm{R}^\mathrm{membrane}_\mathrm{bead}$	$0.5\,\mu\mathrm{m}$
R_cyl, h_cyl		$0.025\,\mu\mathrm{m}$, $0.05\,\mu\mathrm{m}$
Tension	$18k_\mathrm{B}T/(2R^\mathrm{membrane}_\mathrm{bead})^2$	$\sigma = 10\,\mathrm{nN/m}$
Adhesion energy	$8k_\mathrm{B}T$/membrane bead	$W \approx 0.7\,\mu\mathrm{J/m^2}$
Membrane Viscosity		$\eta_\mathrm{eff} \approx 0.8\,\mathrm{Pas}$

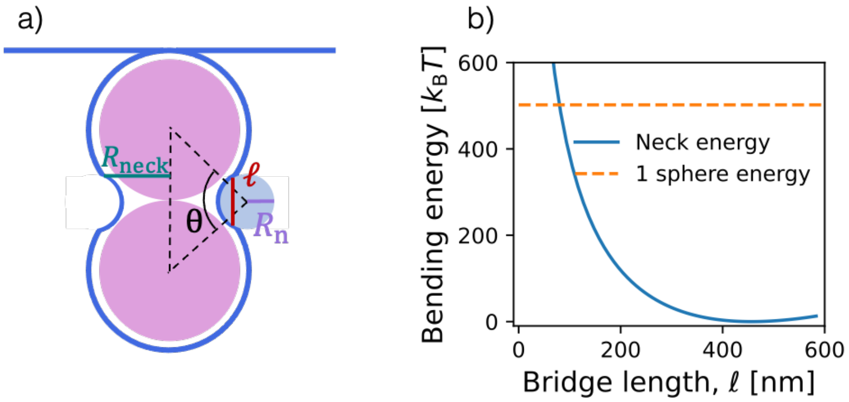

Figure S9: **Estimating the energy barrier for wrapping the full dumbbell from the 1-lobe wrapped state.** The geometry we assume for the energy barrier calculation is shown in a), with the 'bridge length', ℓ, in red. The bending energy barrier is shown in b) for varying bridge lengths. This is compared with the bending energy of one sphere.

tension. We assume that to continue wrapping after the first lobe has already been symmetrically wrapped (the 1-lobe state), the membrane at the surface of the dumbbell must at some point on detach and bridge to the next lobe in order to not take on the high bending cost of wrapping close to the neck region. We constructed an argument in terms of a 'bridge length', ℓ (see Fig. S9a), which we take as the length at which the wrapped 1-lobe membrane may 'see' the next lobe through fluctuations, and can reach the state of two lobes wrapped.

$$\begin{aligned}\mathcal{H}_\mathrm{neck} &= \frac{\kappa}{2}\int_\mathrm{Neck} \mathcal{C}^2 \mathrm{d}A \\ &= \frac{\kappa}{2}\left(\frac{1}{R_\mathrm{n}} - \frac{1}{R_\mathrm{neck}}\right)^2 A\end{aligned} \tag{7}$$

We choose to write all functions and variables in terms of θ, R_{n} and R_{sph} where $\theta = \arccos\left(1 - \frac{2R_{\mathrm{sph}}}{(R_{\mathrm{sph}}+R_{\mathrm{n}})^2}\right)$ is the angle the dumbbell spheres make with the sphere that defines the neck region (see Fig. S9a), R_{n} is the radius of this sphere and R_{sph} is the radius of the dumbbell sphere. The area of the neck region can be written as follows:

$$A = R_{\mathrm{n}}\theta \times 2\pi R_{\mathrm{sph}} \sin\left(\frac{\pi - \theta}{2}\right) \tag{8}$$

Finally, we plot $\mathcal{H}_{\mathrm{neck}}$ as a function of the 'bridge length' which can be written, $\ell = 2\pi R_{\mathrm{n}} \sin\frac{\theta}{2}$.

As is shown in Fig. S9b, the neck energy increases dramatically for a bridge length of less than 200 nm. It reaches a minimum (zero) when the membrane takes on the shape of a catenoid, $R_{\mathrm{neck}} = R_{\mathrm{n}}$. These calculations predict a large energy barrier to reach the two lobe state from the 1-lobe state for membrane fluctuations on the order of less than 100 nm. The energy barrier shown here is a generous upper bound as it assumes the membrane would need to span the whole neck region all at once and in reality the dumbbell probably tilts in a step-wise fashion to wrap the second lobe.

References

(1) Angelova, M. I.; Dimitrov, D. S. Liposome electroformation. *Faraday Discussions of the Chemical Society* **1986**, *81*, 303–311.

(2) Appel, J.; Akerboom, S.; Fokkink, R. G.; Sprakel, J. Facile one-step synthesis of monodisperse micron-sized latex particles with highly carboxylated surfaces. *Macromolecular Rapid Communications* **2013**, *34*, 1284–1288.

(3) Van Der Wel, C.; Bossert, N.; Mank, Q. J.; Winter, M. G.; Heinrich, D.; Kraft, D. J. Surfactant-free Colloidal Particles with Specific Binding Affinity. *Langmuir* **2017**, *33*, 9803–9810.

(4) Lau, A. W.; Prasad, A.; Dogic, Z. Condensation of isolated Semi-flexible filaments driven by depletion interactions. *EPL* **2009**, *87*, 48006.

(5) Allan, D. B.; Caswell, T.; Keim, N. C.; van der Wel, C. M.; Verweij, R. W. *soft-matter/trackpy: Trackpy v0.5.0*, Zenodo. 2021; `https://doi.org/10.5281/zenodo.4682814`.

(6) Van Der Wel, C.; Kraft, D. J. Automated tracking of colloidal clusters with sub-pixel accuracy and precision. *Journal of Physics Condensed Matter* **2017**, *29*.

(7) van der Wel, C. *circletracking v1.0*, Zenodo. 2016; `https://doi.org/10.5281/zenodo.47216`.

(8) Moy, V. T.; Florin, E. L.; Gaub, H. E. Intermolecular forces and energies between ligands and receptors. *Science* **1994**, *266*, 257–259.

(9) Van Der Wel, C.; Vahid, A.; ŠariĆ, A.; Idema, T.; Heinrich, D.; Kraft, D. J. Lipid membrane-mediated attraction between curvature inducing objects. *Scientific Reports* **2016**, *6*, 1–10.

(10) Thompson, A. P.; Aktulga, H. M.; Berger, R.; Bolintineanu, D. S.; Brown, W. M.; Crozier, P. S.; in 't Veld, P. J.; Kohlmeyer, A.; Moore, S. G.; Nguyen, T. D.; Shan, R.; Stevens, M. J.; Tranchida, J.; Trott, C.; Plimpton, S. J. LAMMPS - a flexible simulation tool for particle-based materials modeling at the atomic, meso, and continuum scales. *Computer Physics Communications* **2022**, *271*, 108171.

(11) Yuan, H.; Huang, C.; Li, J.; Lykotrafitis, G.; Zhang, S. One-particle-thick, solvent-free, coarse-grained model for biological and biomimetic fluid membranes. *Phys. Rev. E* **2010**, *82*, 011905.

(12) Agudo-Canalejo, J.; Lipowsky, R. Critical particle sizes for the engulfment of nanoparticles by membranes and vesicles with bilayer asymmetry. *ACS Nano* **2015**, *9*, 3704–3720.

(13) Spanke, H. T.; Agudo-Canalejo, J.; Tran, D.; Style, R. W.; Dufresne, E. R. Dynamics of spontaneous wrapping of microparticles by floppy lipid membranes. *Physical Review Research* **2022**, *4*, 23080.

(14) Frey, F.; Ziebert, F.; Schwarz, U. S. Dynamics of particle uptake at cell membranes. *Physical Review E* **2019**, *100*, 1–18.

(15) Frey, F.; Ziebert, F.; Schwarz, U. S. Stochastic dynamics of nanoparticle and virus uptake. *Physical Review Letters* **2019**, *122*, 88102.

(16) Camley, B. A.; Esposito, C.; Baumgart, T.; Brown, F. L. Lipid bilayer domain fluctuations as a probe of membrane viscosity. *Biophysical Journal* **2010**, *99*, L44–L46.

(17) Hormel, T. T.; Kurihara, S. Q.; Brennan, M. K.; Wozniak, M. C.; Parthasarathy, R. Measuring lipid membrane viscosity using rotational and translational probe diffusion. *Physical Review Letters* **2014**, *112*, 1–5.
